# Magnetoliposomes Containing Multicore Nanoparticles and a New Antitumor Thienopyridine Compound with Potential Application in Chemo/Thermotherapy

**DOI:** 10.3390/biomedicines10071547

**Published:** 2022-06-29

**Authors:** Fábio A. C. Lopes, André V. F. Fernandes, Juliana M. Rodrigues, Maria-João R. P. Queiroz, Bernardo G. Almeida, Ana Pires, André M. Pereira, João P. Araújo, Elisabete M. S. Castanheira, Ana Rita O. Rodrigues, Paulo J. G. Coutinho

**Affiliations:** 1 Physics Centre of Minho and Porto Universities (CF-UM-UP), University of Minho, Campus de Gualtar, 4710-057 Braga, Portugal; pg40847@alunos.uminho.pt (F.A.C.L.); pg38822@alunos.uminho.pt (A.V.F.F.); bernardo@fisica.uminho.pt (B.G.A.); 2 LaPMET (Laboratory of Physics for Materials and Emergent Technologies), Associate Laboratory, 4710-057 Braga, Portugal; ana.pires@fc.up.pt (A.P.); ampereira@fc.up.pt (A.M.P.); jearaujo@fc.up.pt (J.P.A.); 3 Centre of Chemistry (CQUM), University of Minho, Campus de Gualtar, 4710-057 Braga, Portugal; juliana.mourarodrigues@gmail.com (J.M.R.); mjrpq@quimica.uminho.pt (M.-J.R.P.Q.); 4 IFIMUP—Instituto de Física dos Materiais, Universidade do Porto, R. Campo Alegre, 4169-007 Porto, Portugal

**Keywords:** multicore magnetic nanoparticles, magnetoliposomes, magnetic hyperthermia, antitumor thienopyridine derivative, chemotherapy

## Abstract

Multicore magnetic nanoparticles of manganese ferrite were prepared using carboxymethyl dextran as an agglutinating compound or by an innovative method using melamine as a cross-coupling agent. The nanoparticles prepared using melamine exhibited a flower-shape structure, a saturation magnetization of 6.16 emu/g and good capabilities for magnetic hyperthermia, with a specific absorption rate (SAR) of 0.14 W/g. Magnetoliposome-like structures containing the multicore nanoparticles were prepared, and their bilayer structure was confirmed by FRET (Förster Resonance Energy Transfer) assays. The nanosystems exhibited sizes in the range of 250–400 nm and a low polydispersity index. A new antitumor thienopyridine derivative, 7-[4-(pyridin-2-yl)-1*H*-1,2,3-triazol-1-yl]thieno[3,2-*b*]pyridine, active against HeLa (cervical carcinoma), MCF-7 (breast adenocarcinoma), NCI-H460 (non-small-cell lung carcinoma) and HepG2 (hepatocellular carcinoma) cell lines, was loaded in these nanocarriers, obtaining a high encapsulation efficiency of 98 ± 2.6%. The results indicate that the new magnetoliposomes can be suitable for dual cancer therapy (combined magnetic hyperthermia and chemotherapy).

## 1. Introduction

Magnetic nanoparticles (MNPs) have shown to be a promising tool for oncology. Owing to their intrinsic magnetic properties and easy functionalization, they are excellent nanostructures for cancer management, being capable of acting as diagnosis and/or therapeutic agents. In fact, iron oxide nanoparticles have been used in clinical practice as contrast agents for magnetic resonance imaging (MRI) [1]. On the other hand, the therapeutic interest on MNPs relies on the possibility to guide them with a magnetic field gradient to specific tumor locations, enable the controlled release of drugs using drug-loaded MNPs-based nanosystems, and produce local heat under AC magnetic fields (hyperthermia) [2,3]. Advances in nanomedicine have shown promises for magnetic hyperthermia (MHT) and controlled drug delivery using magnetically sensitive nanomaterials. The combination of magnetic hyperthermia with radiation has also shown to enhance tumor control and patient survival rates. In fact, recently, the European Medicines Agency (EMA) has approved NanoTherm^®^ (a colloidal suspension of aminosilane-coated iron oxide NPs with an iron concentration of 112 mg/mL) for MHT application in combination with radiation therapy for patients with recurrent glioblastoma [4]. Yet, despite the most recent advancements, MHT has struggled to establish its clinical presence as a treatment modality for cancer, mainly because of its insufficient heat generation power. Accordingly, the scientific community has been focused on the development of new nanomaterials with enhanced heating performance and biocompatibility to promote its translation for clinical practice.

Cluster nanostructures have shown enhanced heating capabilities when compared to their building blocks, i.e., single nanoparticles. Magnetic nanoclusters with a flower-like structure, consisting of densely packed aggregates of magnetic nanoparticles, have shown enhanced magnetic hyperthermia capabilities, with high specific absorption rate (SAR), evidencing an improved performance as hyperthermia agents [5,6]. In fact, iron oxide nanoflowers have shown superior heating performance when compared to single crystals [7]. The enhanced heating profile of multicore nanostructures has been attributed to the collective magnetic behavior that results from interparticle magnetic interactions (dipole–dipole coupling or exchange coupling) between the cores, which affects the hyperthermia efficiency. Yet, the interparticle interactions are complex and depend on single nanoparticles’ size, orientation and spacing in the aggregates [8]. The preservation of the superparamagnetic behavior by clustered nanostructures (even when their dimension exceeds the superparamagnetic limit) is also an important feature for therapeutic applications, ensuring no magnetization after the removal of the applied AC magnetic field and avoiding agglomeration [9,10,11].

Taking the hyperthermia potential of magnetic nanoflowers and the controlled release possibilities of thermal sensitive liposomes, the development of sensitive magnetoliposomes containing multicore magnetic nanoparticles is of extreme interest, allowing a multifunctional therapeutic approach (combined magnetic hyperthermia and chemotherapy). This promising nanosystem allows the preservation of the magnetic and hyperthermia properties of the multicore nanoparticles [12], while providing suitable drug nanocarriers with increased biocompatibility, flexibility in composition and size, improved drug pharmacokinetics, and prolonged circulation in vivo [13,14]. Particularly, solid magnetoliposomes (nanoparticles covered by a lipid bilayer) containing manganese ferrite nanoparticles have shown similar saturation magnetization as neat nanoparticles, while being especially adequate for the transport of hydrophobic drugs [15]. Furthermore, manganese ferrite nanoparticles have been described as excellent mediators for cancer thermotherapy agents, due to their high magnetic susceptibility and biocompatibility and excellent chemical stability [9,16].

**Figure 1:**
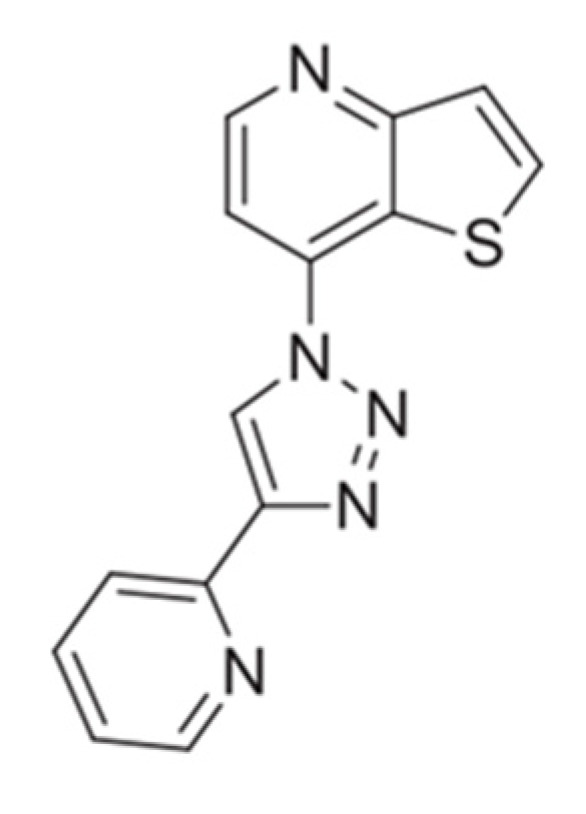
Chemical structure of the new antitumor compound 7-[4-(pyridin-2-yl)-1*H*-1,2,3-triazol-1-yl]thieno[3,2-*b*]pyridine.

In this work, a novel antitumor compound, a thieno[3,2-*b*]pyridine derivative (Figure 1), was encapsulated in magnetoliposomes containing multicore manganese ferrite nanoparticles. This compound has shown promising antitumor activity at very low growth inhibitory concentrations (GI_50_) in four human tumor cell lines, namely, HeLa (cervical carcinoma; GI_50_ =12.99 ± 0.58 µM), MCF-7 (breast adenocarcinoma; GI_50_ =15.13 ± 0.59 µM), NCI-H460 (non-small cell lung carcinoma; GI_50_ = 12.60 ± 0.8 µM) and HepG2 (hepatocellular carcinoma; GI_50_ = 7.51 ± 0.48 µM) [17], being especially active against hepatocellular carcinoma. Moreover, the compound has shown a much lower cytotoxic effect in non-tumor cells PLP2 (Porcine Liver Primary cells; GI_50_ = 91.94 ± 5.23 µM) [17], thus being promising as a chemotherapeutic agent, with no predicted negative impact in normal tissues.

## 2. Materials and Methods

### 2.1. Synthesis of Multicore Manganese Ferrite NPs

The multicore flower-shaped manganese ferrite nanoparticles “A1” and “A2” were obtained using the clustering agent carboxymethyl dextran [18] (Sigma-Aldrich, St. Louis, MO, USA), while for nanoparticles “B”, the aggregation agent melamine (Sigma-Aldrich, St. Louis, MO, USA) was used.

#### 2.1.1. Synthesis Using Carboxymethyl Dextran

**Figure 2:**
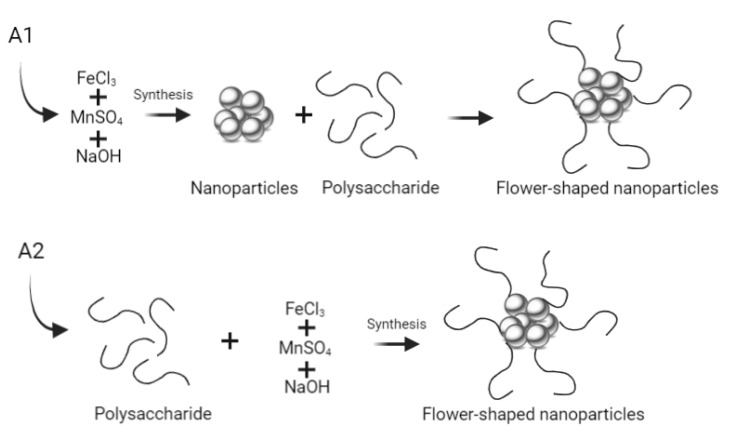
Schematic representation of the process of synthesis of multicore (flower-shaped) NPs using the polysaccharide carboxymethyl dextran.

Two flower-shaped manganese ferrite nanoparticles, A1 and A2, were synthesized using the polysaccharide carboxymethyl dextran as aggregation agent [18]. The synthesis methods differ only in the step of addition of the polysaccharide (Figure 2). In A1 NPs, the synthesis includes a first step consisting in the preparation of the manganese ferrite NPs and a second step consisting in the addition of carboxymethyl dextran to promote NPs aggregation into multicore structures. In A2 NPs, manganese ferrite NPs are prepared in the presence of the polysaccharide.

For the A1 NPs, manganese ferrite (MnFe_2_O_4_) NPs were prepared by coprecipitation, according to a procedure previously described [9]. Briefly, 19 mL of an aqueous solution containing 8 mmol sodium hydroxide (NaOH, 50% in water) was heated to 100 °C. Then, 1 mL of an aqueous solution containing the metallic precursors FeCl_3_·6H_2_O (1.4 mmol) and MnSO_4_·H_2_O (0.7 mmol), both from Sigma-Aldrich (St. Louis, MO, USA), was slowly injected under magnetic stirring. After 15 min, 100 mg of carboxymethyl dextran was added, and the solution was kept at 100 °C, under magnetic stirring, for 2 h.

For the A2 NPs, 19 mL of an aqueous solution containing 8 mmol sodium hydroxide (NaOH, 50% in water) and 100 mg of carboxymethyl dextran was heated up to 100 °C. Then, 1 mL of the metallic precursors solution (1.4 mmol of FeCl_3_·6H_2_O and 0.7 mmol of MnSO_4_·H_2_O) was added, and the solution was kept, under magnetic stirring, at 100 °C, for 2 h. Finally, the obtained A1 and A2 NPs were washed with water and ethanol, with several steps of washing and magnetic decantation.

#### 2.1.2. Synthesis Using Melamine

The flower-shaped NPs B were prepared using the clustering agent melamine. In this method, the reagents *N*,*N*′-carbonyldiimidazole (CDI) from FluoroChem (Derbyshire, UK), melamine and imidazole from Sigma-Aldrich (St. Louis, MO, USA) were used [5,6].

**Figure 3:**
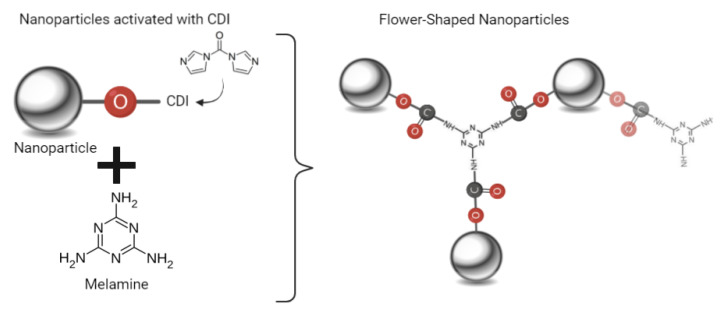
Schematic representation of the synthesis of flower-shaped NPs B, using melamine.

First, manganese ferrite NPs were obtained as previously described [9]. Then, the −OH groups of the NPs surface were activated before the addition of the clustering agent. For that, 4.3 × 10^−5^ mol of the prepared manganese ferrite NPs were dispersed in 7 mL of dry dimethyl sulfoxide (from Sigma-Aldrich, St. Louis, MO, USA). Then, 2.2 × 10^−4^ mol of CDI was added, and the solution was kept at 60 °C, under sonication. After 2 h, ultrapure water was used to eliminate the excess of CDI, and then, 4.3 × 10^−5^ mol of melamine was added. Finally, an equivalent quantity of imidazole (4.3 × 10^−5^ mol) was added, and the reaction was kept at 60 °C, for 2 h, under sonication. Imidazole was used to promote a faster coupling between the –NH_2_ groups of melamine and the –OH groups on NPs surface (Figure 3) [19,20].

### 2.2. Magnetoliposomes Preparation

Magnetoliposome-type structures were prepared following a previously described procedure for the preparation of liposomes based on manganese ferrite/gold NPs [21]. Accordingly, 1 × 10^−6^ mol of multicore manganese ferrite nanoparticles were dispersed in 5 mL of ethanol, and octadecylamine (ODA, from Sigma-Aldrich, St. Louis, MO, USA) was added in a 5-fold excess. After 1 h under magnetic stirring at a temperature of 60 °C, the solution was washed in successive magnetic decantation/centrifugations steps, to remove the non-bound octadecylamine. A 1 mM solution of dipalmitoylphosphatidylcholine (DPPC, from Sigma-Aldrich, St. Louis, MO, USA) in ethanol was added to the nanoparticles covered with ODA. After solvent evaporation, under an ultrapure nitrogen stream, a uniform film (consisting of ODA-covered NPs and DPPC) was obtained. Finally, 5 mL of ultrapure water Milli Q-grade (from MilliporeSigma, St. Louis, MO, USA) was added to this film, followed by sonication. Then, two washing steps were carried out, consisting of centrifugation/magnetic decantation, for the purification of the prepared magnetoliposomes.

**Figure 4:**
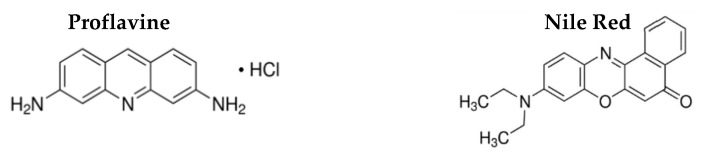
Chemical structure of the Proflavine and Nile Red probes.

The formation of liposome-like structures was confirmed by FRET (Förster Resonance Energy Transfer) measurements. For that, two fluorescent probes, proflavine (acting as the energy donor) and Nile Red (as the energy acceptor), both from Sigma-Aldrich, (St. Louis, MO, USA), were employed (the structures of the fluorescent probes are shown in Figure 4). The fluorescence dye proflavine was coupled to the flower-shaped NPs surface, right before the formation of the ODA layer, taking advantage of NPs surface activation by CDI, and Nile Red was included together with the DPPC layer formation.

FRET efficiency (Φ_FRET_) and the distance between the donor molecules and the acceptor ones ($r_{AD}$) were calculated as previously reported [9,21], according to Equations (1)–(4) [22],

(1) $$\Phi_{\mathrm{FRET}}=1-\frac{F_{DA}}{F_{D}}$$

where *F_DA_* and *F_D_* represent the donor-integrated fluorescence intensities in the presence of the acceptor and in the absence of the acceptor, respectively;

(2) $$r_{AD}=R_{0}\;{\left[\frac{\;1-{\;\Phi }_{\mathrm{FRET}}\;}{\Phi_{\mathrm{FRET}}}\right]}^{1/6}$$

where *R*_0_ is the Förster radius, obtained through the spectral overlap, *J*(*λ*), between the donor emission and the acceptor absorption, according to the following relations (Equations (3) and (4), where $R_{0}$ is in Å, *λ* is expressed in nm, $\varepsilon_{A}\left(\lambda \right)$ in M^−1^ cm^−1^):

(3) $$R_{0}=0.2108\;{\left[k^{2\;}\Phi_{D\;}^{0}n^{-4}\;J\left(\lambda \right)\right]}^{1/6}$$

(4) $$J\;\left(\lambda \right)=\int_{0}^{\infty }I_{D}\left(\lambda \right)\varepsilon_{A}\left(\lambda \right)\lambda^{4}d\lambda$$

In these equations, *k*^2^ = 2/3 is the orientational factor with random orientation of the dyes used, $\Phi_{D\;}^{0}$ represents the donor fluorescence quantum yield in absence of the energy transfer process, *n* is the refraction index of the medium used in the sample, $I_{D}\left(\lambda \right)$ is the donor fluorescence spectrum normalized to obtain $\int_{0}^{\infty }I_{D}\left(\lambda \right)d\lambda =1$, and $\varepsilon_{A}\left(\lambda \right)$ is the acceptor molar absorptivity.

The fusion ability of the prepared magnetoliposome-like structures with model membranes was assessed. In this assay, as model membranes, giant unilamellar vesicles (GUVs) were employed. GUVs of lecithin from soybean (Sigma-Aldrich, St. Louis, MO, USA) were obtained using a protocol described elsewhere [23]. Briefly, a thin film of 1 mM of soy lecithin (1 mM) was pre-hydrated with 80 µL of ultrapure water and incubated at 45 °C, for 30 min. After that, 6 mL of a 6 × 10^−4^ M glucose aqueous solution was added, and this mixture was kept at 37 °C for 2 h. Finally, the solution was centrifuged for half an hour at 10,000 rpm, and then the supernatant was collected. This procedure guarantees the removal of lipid aggregates within the pellet.

### 2.3. Sedimentation Curves of the Nanoparticles

The sedimentation profile of NPs suspensions is crucial to determine their colloidal stability. The absorption of the nanoparticles suspensions (concentrations of 0.025%, 0.05% and 0.2% *m/v*) was determined for 1 h, and the experimental data were fitted to the Becquerel’s decay function, given by Equation (5)

(5) It=11+ctτ01/c

where the control parameter *c* varies in the range 0 < *c* < 1, and $\tau_{0}$ has time dimensions [24].

### 2.4. Techniques for Sample Characterization

The magnetic characterization of the NPs was performed in a MPMS3 SQUID magnetometer MPMS5XL (Quantum Design Inc., San Diego, CA, USA), using applied magnetic fields up to 5 T. The hysteresis cycles were obtained by measuring the magnetization in a series of different applied magnetic fields, at room temperature.

The nanostructures images were recorded using a Scanning Electron Microscopy, model NanoSEM-FEI Nova 200 (FEI Technologies, Inc., Hillsboro, OR, USA), in transmission mode (STEM). The software *ImageJ* (from National Institutes of Health (NIH), version 1.53c, Bethesda, MD, USA) was utilized to process the experimental microscopy images by increasing contrast and subtracting background.

The hydrodynamic diameter, zeta potential value and polydispersity were measured by Dynamic Light Scattering, using a NANO ZS Malvern Zetasizer (Malvern Panalytical Ltd., Malvern, UK) apparatus with a He-Ne laser (*λ* = 632.8 nm). For each sample to be characterized, five independent measurements were performed.

### 2.5. Photophysical Study of the Antitumor Compound in Solution

The absorption and fluorescence emission properties of the antitumor thienopyridine derivative were studied in different solvents, namely ethyl acetate, ethanol and acetonitrile (from Sigma-Aldrich, St. Louis, MO, USA). In all preparations, spectroscopic-grade solvents and ultrapure deionized water of Milli-Q grade (MilliporeSigma, St. Louis, MO, USA) were used. The UV–Vis–NIR spectrophotometer Shimadzu UV-3600 Plus (Shimadzu Corporation, Kyoto, Japan) was used to determine the absorption spectra, and the spectrofluorimeter Fluorolog 3 (HORIBA Jobin Yvon IBH Ltd., Glasgow, UK) was utilized to measure the fluorescence emission spectra.

The fluorescence quantum yields of the compound were determined using a quinine sulfate solution (1 ppm in 0.05 M sulfuric acid, $\Phi_{r}$ = 0.546 at 25 °C) as the reference [25], following the classic standard method, according to Equation (6) [26,27],

(6) $$\Phi_{S}=\left[\frac{A_{r}F_{S}n_{S}^{2}}{A_{S}F_{r}n_{r}^{2}}\right]\Phi_{r}$$

taking A as the absorbance value at the excitation wavelength, F as the integrated emission spectral area, and n as the refractive index of the solvent used. The subscript *r* refers to the reference, and the subscript *s* to the sample. Prior to these measurements, the solutions were deaerated by a stream of ultrapure nitrogen for half an hour.

### 2.6. Encapsulation Efficiency of the Compound in the Magnetoliposomes

The novel antitumor compound, a thienopyridine derivative, was encapsulated into the magnetoliposomes containing multicore manganese ferrite NPs. For that, the compound (5 × 10^−^^5^ M) in ethanol was added together with the lipid DPPC to the ODA-covered NPs for thin film formation, as explained previously in Section 2.2.

The encapsulation efficiency, EE(%), of the antitumor compound in the magnetoliposomes was obtained using Amicon^®^ Ultra centrifugal filter units of 100 kDa (Merck Millipore, Darmstadt, Germany) for the separation of the encapsulated and non-encapsulated compound. Compound-loaded nanostructures were subjected to a 10 min centrifugation, at 3000 rpm, and the fluorescence of the non-encapsulated compound was measured. The fluorescence intensity was converted into concentration using a previously obtained calibration curve. Finally, the EE (%) was determined using Equation (7),

(7) $$\mathrm{EE}\left(\%\right)=\frac{C_{\left(total\;compound\right)}-C_{\left(non-encapsulated\;compound\right)}}{C_{\left(total\;compound\right)}}\times 100$$

### 2.7. Magnetic Hyperthermia

The heating and cooling curves were obtained using a magneTherm equipment from nanoTherics (Warrington, UK), using three different frequencies (*f* = 161 kHz, 270 kHz, and 381 kHz) and two field amplitudes (*H* = 16 mT and 17 mT). Before starting the measurement, the temperature of the sample was stabilized, and then an alternating field was applied, while the temperature was recorded for a period of 30 min. Afterwards, the applied field was turned off, and the cooling of the samples was recorded for 30 min. These tests allowed the calculation of the specific absorption rate (SAR) and the intrinsic loss power (ILP), two parameters that indicate the ability of the NPs to generate heat in the presence of an external magnetic field. The SAR is defined as the amount of energy absorbed by the sample, per unit of mass (W/g), and can be obtained by Equation (8)

(8) $$\mathrm{SAR}=C\times \frac{\Delta T}{\Delta t}\times \frac{m_{s}}{m_{m}}$$

where C is the specific heat capacity of the suspension, $\frac{\Delta T}{\Delta t}$ is the initial slope of the curve, and $m_{s}$ and $m_{m}$ express the mass of the suspension and the magnetic material content in suspension, respectively.

The intrinsic loss power (ILP, nH.m^2^/kg) was obtained by Equation (9)

(9) $$\mathrm{ILP}=\frac{SAR}{H^{2}f}$$

where *H* is the field strength in kA/m, and *f* is the frequency in kHz [28].

## 3. Results and Discussion

### 3.1. Characterization of the Multicore Nanoparticles

**Figure 5:**
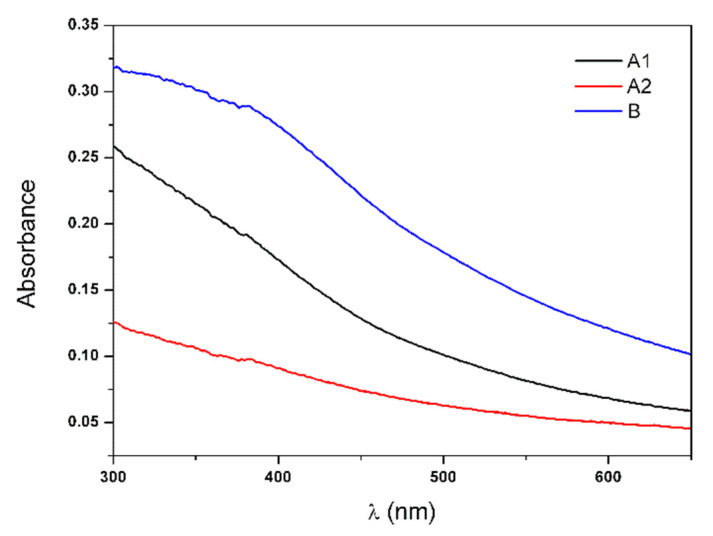
UV–visible absorption spectra of aqueous dispersions of multicore nanoparticles A1, A2 and B.

The produced multicore NPs were characterized by different techniques to evaluate their size, shape, stability, magnetic properties and heating capabilities. The UV–Visible absorption spectra of aqueous dispersions of nanoparticles A1, A2 and B are shown in Figure 5.

A wide-range absorption was observed for the nanoparticles A1, A2 and B, with an absorption band around 400 nm. This band was more prominent for NPs B, showing an increased intensity in comparison with the band of NPs A1 and A2. In addition, the attenuation observed in light scattering in the range between 300 nm to 400 nm, compared to that for NPs A1 and A2, allowed anticipating a different structural organization within the multicore nanoparticles depending on the use of melamine or dextran as aggregation agents.

The structures obtained from the different methods can be a determining factor for their colloidal stability. The sedimentation rates (*k*) of the aqueous NPs dispersions were measured for three different concentrations, 0.025% (*m/v*), 0.05% (*m/v*), and 0.2% (*m/v*). The results are shown in Table 1.

In general, all NPs were stable, presenting small sedimentation rates which increase with NPs concentration. NPs A1 and B displayed a stronger rate dependence on the concentration, showing that the use of the clustering agent after NPs synthesis may result in larger nanostructures. The lower rate observed for sample A2 indicates that the addition of the polysaccharide during the synthesis process of the nanoparticles improves their colloidal stability.

**Figure 6:**
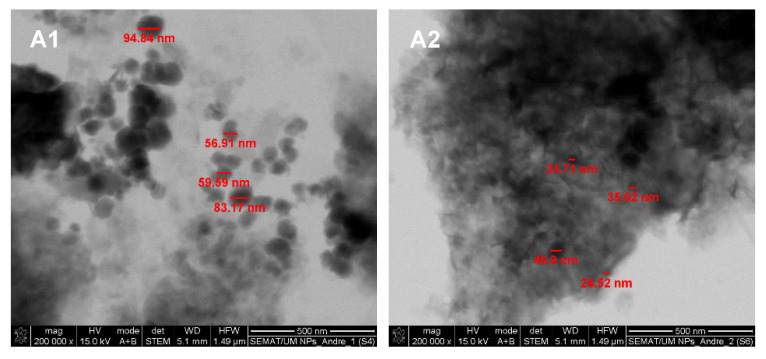
STEM images of multicore NPs from samples A1 and A2.

**Figure 7:**
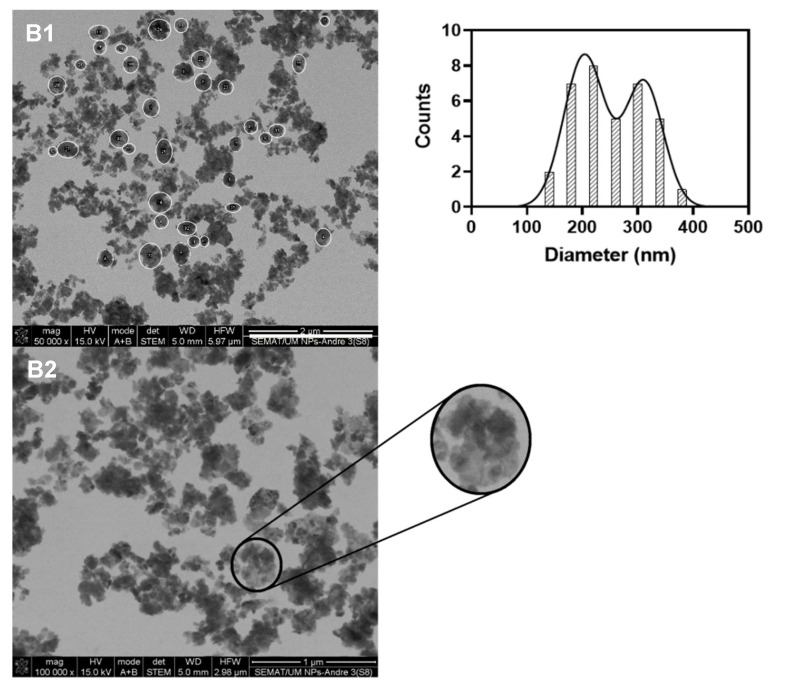
SEM images of sample B at different magnifications. (B**1**) Manual identification of various flower-like structures and corresponding size histogram fitted to the sum of two Gaussian distributions (right panel). (B**2**) Identification and enlargement of a flower-shaped structure.

The size and shape assessment of the multicore manganese ferrite NPs was performed by SEM (in transmission mode, STEM). Despite the small size of the nanoparticles and agglomeration blare, it was possible to observe a generally spherical shape of the NPs (Figure 6 and Figure 7).

The SEM images revealed different types of structures for NPs A1 and A2 (Figure 6). NPs A1 appeared larger in size, with diameters between 59.5 nm and 95 nm. On the other hand, NPs A2 were smaller, with sizes varying from 24.5 nm to 47 nm. It was also possible to observe that the addition of the polysaccharide carboxymethyl dextran after MnFe_2_O_4_ NPs synthesis (NPs A1) did not promote the formation of multicore NPs, while its presence during MnFe_2_O_4_ NPs synthesis (NPs A2) promoted their integration into an organic matrix. Yet, disorganized structures without standard size or shape were obtained using the polysaccharide carboxymethyl dextran.

On the other hand, the use of melamine as a cross-linking agent (NPs B) resulted in multicore structures with a generally spherical shape (Figure 7). The size distribution of these structures was obtained using *ImageJ* software. For that, the flower-shaped structures were manually selected (35 counts, Figure 7 B1), and their corresponding area was converted into diameters. The results were adjusted to the sum of two Gaussians, allowing obtaining a large size distribution of 203 ± 37 nm and 311 ± 35 nm (Figure 7). In fact, large size aggregates with poor internal order in aqueous media were reported [29]. Yet, NPs with a hydrodynamic diameter between 100 and 400 nm have been considered optimal for passive tumor targeting due to the enhanced permeability and retention (EPR) effect [30]. Hence, NPs B are suitable candidates to act as localized thermal agents for tumor treatment.

**Figure 8:**
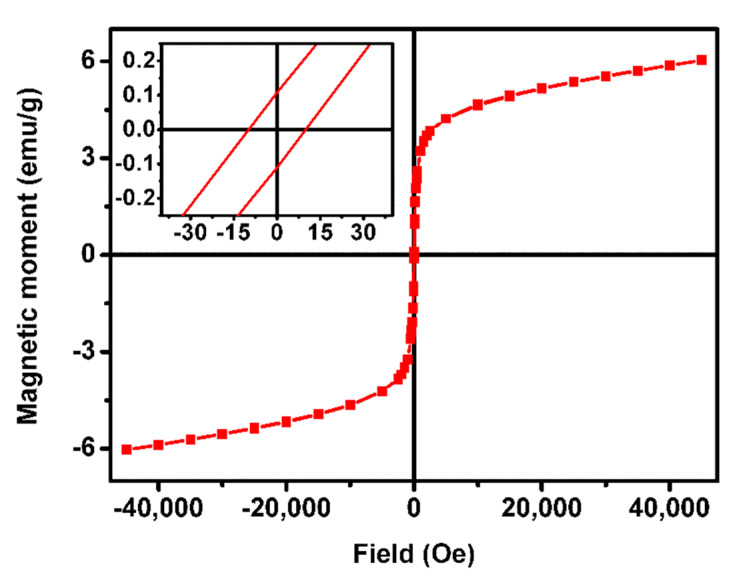
Hysteresis curve of the multicore manganese ferrite NPs B, at room temperature. Insert: Magnification of the low-field zone of the hysteresis curve.

Considering the SEM results, multicore NPs B were selected for magnetic characterization and magnetoliposomes preparation. The magnetic properties of the nanoparticles were evaluated in a SQUID equipment, and the obtained hysteresis loop is displayed in Figure 8. The values of coercivity, remnant magnetization and saturation magnetization and the ratio between remnant and saturation magnetization are summarized in Table 2.

The magnetic behavior of multicore NPs is complex and depends on the sizes of the primary NPs and the final aggregated structure, which can result in random cores or well-oriented ones. In addition, the strength of the magnetic interactions between the primary NPs and their spatial arrangement plays an important role in determining the magnetic properties of this type of nanostructures [29]. The synthesized multicore NPs B showed a superparamagnetic behavior at room temperature, presenting a remnant and saturation magnetization ratio below 0.1, the superparamagnetic limit in which more than 90% of the magnetization is lost after removal of the applied field [31]. Small hysteresis was obtained with a coercivity of 16.23 Oe and a remnant magnetization of 0.11 emu/g. The poor saturation magnetization of 6.16 emu/g obtained can be attributed to different phenomena that have been proposed to explain the unusual behavior of multicore NPs. Possible explanations include the coupling of the spins of the cores within the multicore NPs, leading to finite effective moments [32,33], and frustrated dipole–dipole interactions of the clusters that can reduce the magnetic moment. On the other hand, the non-close packed arrangement between the core NPs within the multicore nanostructures leads to predominant dipolar interactions over exchange energy, which increase NPs’ susceptibility and magnetization. Hence, the large size aggregates with poor internal order are consistent with the low saturation magnetization obtained.

**Figure 9:**
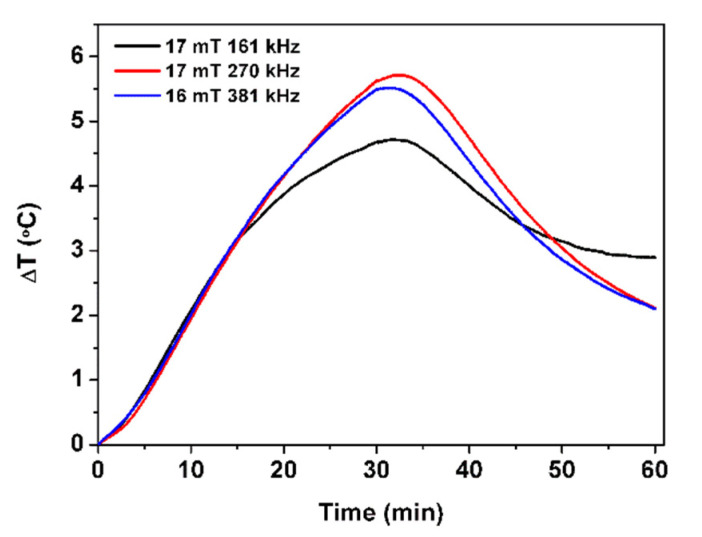
Temperature variation of NPs B, in the presence of alternating magnetic fields with amplitude of 17 mT and frequencies of 161 kHz and 270 kHz and amplitude of 16 mT and frequency of 381 kHz.

The heating ability of the multicore NPs B was evaluated under a high-field and -frequency alternating magnetic field (AMF), with three different field and frequency conditions (17 mT and 161 kHz, 17 mT and 270 kHz, or 16 mT and 381 kHz). Before applying the AFM, the temperature of the sample was stabilized. The heating and cooling curves obtained are shown in Figure 9.

Taking the mild hyperthermia range between 40 °C and 43 °C and the body temperature around 37 °C, a local increase of 5 °C should be strong enough for an effective therapeutic effect. In a time of 30 min, temperature variations up to 4.7 °C (17 mT and 161 kHz), 5.5 °C (16 mT and 381 kHz) and 5.7 °C (17 mT and 270 kHz) were obtained, corroborating the potential of the multicore NPs B as hyperthermia agents. Despite presenting poor saturation magnetization, the good heating capability of NPs B is attributed to their flower-like structure. In fact, a similar behavior has already been reported for iron oxide nanoclusters aggregates with carboxymethyl dextran [34]. Table 3 displays the SAR and ILP values of NPs B, calculated in the three experimental conditions. The higher SAR value of 0.14 W/g of neat NPs B (mass of MnFe_2_O_4_) was obtained under the alternating magnetic field of 16 mT and 381 kHz. On the other hand, the higher ILP value (0.46 nH.m^2^/kg) was obtained for the NPs B subjected to the magnetic field of 17 mT with a frequency of 161 kHz. ILP values between 0.1 and 0.58 have been reported for manganese-doped spherical ferrites [35]. Thus, the values obtained for NPs B are in accordance with the ones reported for this type of NPs. It is concluded that the NPs produced have suitable characteristics for application in magnetic hyperthermia therapeutic approaches, while their superparamagnetic behavior will avoid their aggregation when the applied magnetic field is removed [10,34].

### 3.2. Magnetoliposomes Characterization

**Figure 10:**
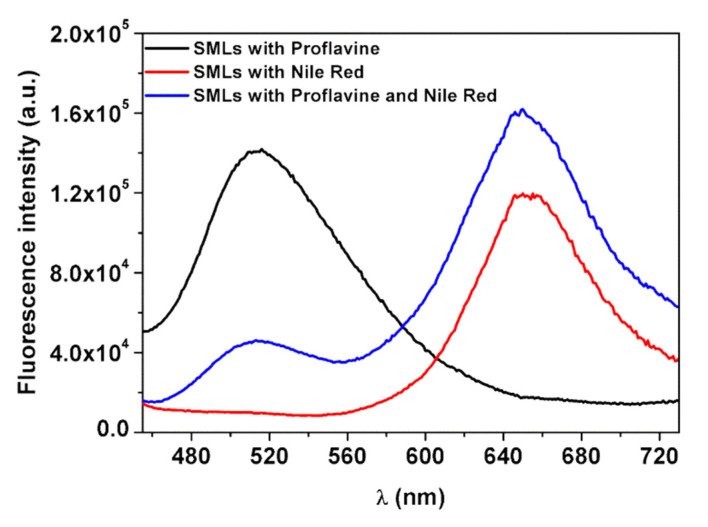
Fluorescence spectra of MLs (based on NPs B) containing only proflavine (5 × 10^−6^ M, λ_exc_ = 380 nm), only Nile Red (5 × 10^−6^ M; λ_exc_ = 450 nm) or both fluorophores (5 × 10^−6^ M each, λ_exc_ = 380 nm).

Considering the potential of multicore NPs B, magnetoliposomes (MLs) based on these nanostructures were prepared. Förster resonance energy transfer (FRET) was used to prove the formation of the double layer of MLs membrane. For that, proflavine was used as an energy donor (being included before adding the first ODA layer), while the hydrophobic dye Nile Red, as an energy acceptor, was incorporated within the outer lipid layer. MLs with only proflavine or with only Nile Red were also prepared. The emission of the MLs was measured by exciting only the donor (proflavine), and the obtained spectra are shown in Figure 10.

Comparing the emission of proflavine from MLs loaded with only proflavine and the systems containing proflavine and Nile Red, a decrease in the proflavine emission band (at ~510 nm) was detected. At the same time, when observing Nile Red emission (at ~660 nm), an increase in fluorescence intensity was detected for the MLs loaded with both proflavine and Nile Red, compared to the MLs loaded with only Nile Red, indicating that proflavine-excited molecules transferred their excitation energy to Nile Red. Thus, the increase in emission of the acceptor band and the consequent decrease of the donor confirmed the energy transfer between the two fluorescent probes. FRET efficiency, Förster radius and the distance between donor and acceptor (Equations (1)–(4)) were calculated, and these results are displayed in Table 4. A high FRET efficiency of 68% was obtained, for a corresponding donor–acceptor distance of 1.2 nm.

The size of a typical phospholipid bilayer is of 4.5 nm, similar to that of the liposome-like structure composed of ODA and DPPC in magnetoliposomes [36]. Considering that FRET only occurs at a donor–acceptor distances below 10 nm and that proflavine molecules were located at the flower-shaped structures surface, while the hydrophobic dye Nile Red was located within the bilayer of the liposome-like structures, this donor–acceptor distance supports the formation of a bilayer around the flower-shaped NPs and, consequently, the synthesis of magnetoliposomes [15,21].

**Figure 11:**
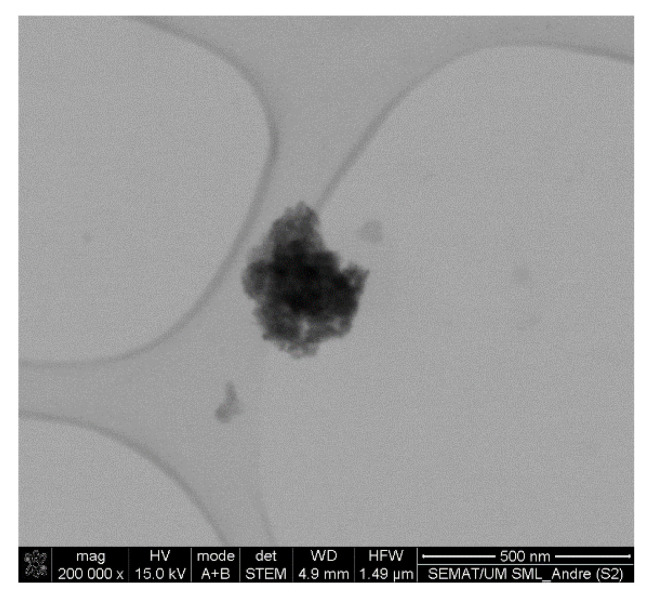
SEM images of magnetoliposomes based on NPs B.

The colloidal stability of the magnetoliposomes based on NPs B (with a concentration of 0.025 % *m*/*v*) was studied, and their structural characterization was performed by DLS (Table 5) and SEM (Figure 11). A sedimentation rate of 0.0014 min^−1^ for MLs based on NPs B was obtained. This value is slightly smaller compared to the rate obtained for the net NPs B at the same concentration (Table 1), indicating that their encapsulation in liposomes promoted colloidal stability. The mean values of hydrodynamic diameter, polydispersity (PDI) and zeta potential are summarized in Table 5.

Hydrodynamic diameters of 388 ± 22 nm were obtained, indicating that the synthesized liposomes were able to encapsulate single flower-like nanostructures. A polydispersity index value of 0.2 ± 0.11 was measured, showing an MLs homogeneous population. The neutral surface charge (−2.4 ± 7.4 mV) is in accordance with the expected value, given the zwitterionic charge of the outer lipid DPPC. SEM images enabled the visualization of MLs with a spherical shape and diameters around 300 nm (Figure 11). Overall, the SEM results agree with the DLS data, considering that DLS measures hydrodynamic diameters (while in SEM, samples are dried).

### 3.3. Drug-Loaded Magnetoliposomes

**Figure 12:**
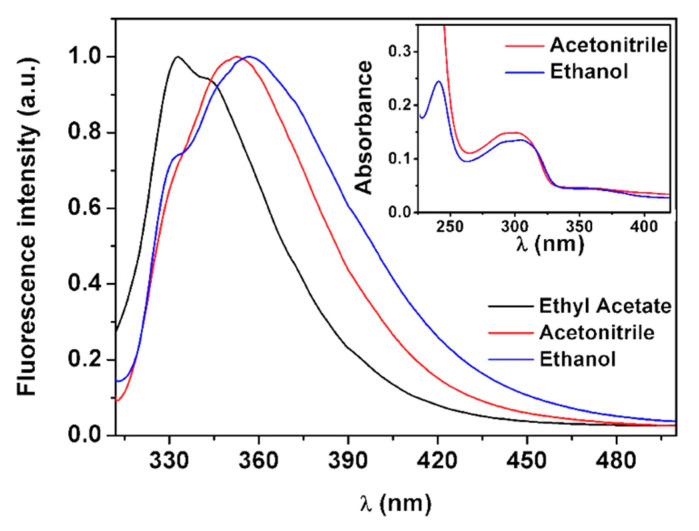
Normalized fluorescence spectra of the antitumor thienopyridine derivative (5 × 10^−6^ M) (λ_exc_ = 300 nm) in solvents of different polarity. Inset: Absorption spectra of the compound (1 × 10^−5^ M) in ethanol and acetonitrile.

The new antitumor thienopyridine derivative was loaded into the magnetoliposomes based on NPs B. Before its encapsulation, the photophysical properties of the compound were studied. For that, the UV–Visible absorption and fluorescence spectra in several solvents with different polarities were measured (Figure 12).

The antitumor compound demonstrated to be fluorescent in all solvents, with fluorescence quantum yields of 3% and 4%. A general redshift of the emission band with increasing polarity was detected [22]. This behavior is similar to that observed for other thieno[3,2-*b*]pyridine-based compounds with antitumor and/or antiangiogenic properties, previously synthesized and studied [37,38,39,40,41]. The maximum absorption and emission wavelengths, molar absorption coefficients and fluorescence quantum yields are shown in Table 6.

A high encapsulation efficiency (EE%) is a key in the development of a drug nanocarrier. The EE% of the antitumor compound loaded in the MLs was determined to be 98 ± 2.6% (using Equation (7)). The high EE% obtained indicates that these MLs are excellent encapsulation systems for this antitumor compound, being promising to endorse its targeted delivery by means of external magnetic fields and controlled drug delivery by application of an alternating magnetic field.

## 4. Conclusions

Multicore flower-like manganese ferrite nanostructures with size distributions of 203 ± 37 nm and 311 ± 35 nm were obtained by an innovative method using melamine as a cross-linking agent. Despite their low saturation magnetization of 6.16 emu/g, their great heating capabilities, with a SAR value of 0.14 W/g (corresponding to an ILP of 0.22 nH.m^2^/kg), point to their promising use as magnetic hyperthermia agents. The synthesis of liposome-like structures based on these multicore nanoparticles was confirmed by FRET assays, using proflavine as an energy donor and the lipid probe Nile Red as an acceptor. A new antitumor fluorescent compound, 7-[4-(pyridin-2-yl)-1*H*-1,2,3-triazol-1-yl]thieno[3,2-*b*]pyridine, active against HeLa, MCF-7, NCI-H460 and HepG2 tumor cells, was efficiently loaded within the nanosystem, with 98% ± 2.6% encapsulation efficiency. Hence, these magnetoliposomes are promising nanocarriers for the combination of chemotherapy and magnetic hyperthermia in cancer treatment.

## Figures and Tables

**Table 1:** Sedimentation rates (*k*) calculated by the Becquerel function for NPs A1, A2 and B.

Concentration (% *m*/*v*)	*k* (min^−1^)		
	NPs A1	NPs A2	NPs B
0.025	0.0011	0.0008	0.0017
0.05	0.0016	0.0008	0.0017
0.2	0.0034	0.0009	0.0021

**Table 2:** Values of coercivity, remnant magnetization and saturation magnetization and ratio between remnant magnetization and saturation magnetization (M_r_/M_s_), obtained from the hysteresis curve.

	Coercivity (Oe)	Remnant Magnetization (emu/g)	Saturation Magnetization (emu/g)	M_r_/M_s_
NPs B	16.23	0.11	6.16	0.02

**Table 3:** SAR and ILP values, obtained from the heating and cooling curves of NPs B.

	17 mT, 161 kHz	17 mT, 270 kHz	16 mT, 381 kHz
SAR (W/g)	0.12	0.10	0.14
ILP (nH.m^2^/kg)	0.46	0.20	0.22

**Table 4:** Förster resonance energy transfer parameters, FRET efficiency (Φ_FRET_), Förster radius (*R*_0_) and donor–acceptor distance (*r*) obtained.

	Φ_FRET_	*R*_0_ (Å)	*r* (Å)
MLs	0.68	13.6	12

**Table 5:** Mean values and standard deviation of hydrodynamic diameter, polydispersity index (PDI) and zeta potential of magnetoliposomes based on multicore NPs, obtained by DLS.

	Hydrodynamic Diameter (nm)	PDI	Zeta Potential (mV)
MLs NPs B	388 ± 22	0.2 ± 0.11	−2.4 ± 7.4

**Table 6:** Maximum absorption wavelengths (λ_abs_), molar absorption coefficients (ε), maximum emission wavelengths (λ_em_) and fluorescence quantum yields (Φ_F_) of the antitumor compound.

Solvent	λ_abs_/nm (ε/10^4^ M^−1^ cm^−1^)	λ_em_ (nm)	Φ_F_
Ethyl acetate	288 (1.1)	333	0.04
Acetonitrile	301 (0.7)	353	0.03
Ethanol	305 (0.7)	357	0.03

## Data Availability

Not applicable.
